# Cognitive Beliefs and Future Time Perspectives: Predictors of Mortality and Longevity

**DOI:** 10.4061/2011/367902

**Published:** 2011-10-03

**Authors:** Prem S. Fry, Dominique L. Debats

**Affiliations:** ^1^Graduate Psychology Program, Trinity Western University, 7600 Glover Road, Langley, BC, Canada V2Y 1Y1; ^2^Groningen, The Netherlands

## Abstract

On the basis of postulates derived from cognitive-behavioral theory, research and therapy, the authors explored the extent to which older adults' cognitive beliefs of a just world and their perspectives on future time and similarity or self-continuity with the future self are predictors of long-term survival. After baseline assessment of health and cognitive beliefs and future perspectives of time and self-continuity as predictors of mortality, 440 participants (ages 65 to 87) were followed longitudinally for 6.5 years. Consistent with our hypotheses, findings demonstrated that a significantly higher percentage of survivors were individuals who showed higher scores on beliefs in a just world and on both the future time perspective and the future self-continuity perspective at the time of baseline assessments. Conversely, mortality risk was much higher for individuals who scored low on these predictor variables, and high on distrust. Implications for health and longevity are discussed.

## 1. Introduction

To date, one of the most understudied etiologies of older adults' survival and longevity has been the role of their cognitive beliefs and worldviews that possibly interact with their functional and mental capacities to endure, challenge, overcome, and survive in the face of the numerous struggles and obstacles in advanced old age. 

In earlier research on predictors of mortality, the focus has been exclusively on variations in physical health and sociodemographic variables to explain and predict differences in longevity and mortality rates across a wide age range. More recently, studies have explored the relationship between the 5-factor personality traits [1–4] and other stress-inducing traits of perfectionism and dysfunctional dependency traits to predict greater longevity or increased risk of mortality [5]. The present study presents a clear departure from earlier studies that have focused on sociodemographic and personality factors to explain and predict differences in all-cause mortality rates in later life. The goal of the present research is to move outside the personality and traits model to other second-order cognitive-behavioral factors to predict differences in all-cause mortality rates in later life. Cognitive-behavioral theorists argue on both theoretical and empirical grounds that individuals' cognitive beliefs exert a great deal of influence on their health, resilience, and longevity and may logically be assumed to be robust predictors of impending mortality or conversely of longevity. However, the ability of cognitive belief systems to predict important health outcomes of survival and longevity has traditionally been questioned because of the putative effects of individuals' earlier life experiences such as parental loss and divorce [6]. More recent explorations into individuals' cognitive beliefs have been drawing attention to a cluster of beliefs systems that may counter the effects of earlier negative experiences and may serve as strengthening factors toward enhancing longevity. Recent research using more modern concepts of evaluating dominant cognitive beliefs and cognitive perspectives of individuals (e.g., beliefs about a just world (BJW) for self and others, beliefs about one's future time, beliefs about one's self-continuity with the future, and beliefs about social, political, and interpersonal trust) has provided growing evidence that individuals' cognitive beliefs and perspectives are indeed related to health-related processes [7, 8] leading to longer survival and longevity as a final health outcome. Also, in recent years, the availability of reliable and valid measures of cognitive beliefs of justice and fairness [9, 10], future time perspectives [11], and future self-continuity perspectives [12, 13] has increased our understanding of the predictive value of individuals' cognitive beliefs as strengthening or debilitating factors in health-related outcomes of resilience and longevity.

The present study addresses the relationship between newly emerging sets of important cognitive belief systems and the potential for increased longevity. For example, a number of researchers [10, 14] have demonstrated empirically that individuals' tenacious cognitive beliefs in a just world (BJW) society are not only predictive of their subjective well being and resilience, but more importantly drive them toward investment in long-term goals and a commitment to better self-care of health and a longer life. As a result, life-span scholars are now more keenly exploring the proactive role that individuals' beliefs about a just world (BJW) may play in their future well being and healthy physical survival (see [15] Tomaka and Blascovitch, 1994), low levels of depression [16], and less loneliness [17]. Other dominant cognitive beliefs which have been seen to be related to healthy survival processes or which present increased risks of mortality include beliefs about interpersonal trust and trust in key institutions [18, 19]. Individuals who have strong positive beliefs of trust in the interpersonal and institutional domains are commonly expected to live lives that are more organized and planned, as distinguished from lives of instability, anxiety, and caution [19]. Other perspectives and belief systems that are predictive of planned healthy survival include “Future Time Perspective (FTP)” and “Self-Continuity with the Future Perspective” (FSC). The FTP perspective is a measure of individuals' perceived belief about how much time participants had left in life. According to Carstensen [11], the subjective sense of remaining time has profound effects on basic human processes, including motivation, cognition, and behaviors. With increasing age, constraints on time left shift individuals' priorities about how remaining time can be protected. Along somewhat similar lines, the future self-continuity perspective (FSC) indicates that participants' beliefs about their similarity and connection as well as caring and liking for their future self 10-, 15-, or 20-years from now [13] determine and shift their motivation to protect the potential future person. For purposes of the present study, our underlying conceptual assumption in both these futuristic perspectives is that individuals who perceive their time horizons and their self-continuity with the future as more limited would be more likely to discount the future, and thus more unlikely to plan for self-care and self-management of the future, whereas those who feel the future horizons are more open-ended and expansive are more likely to plan and organize for a secure future. Implicit in these perspectives is the prediction that individual differences in the experience of self-continuity could have positive pragmatic consequences for future health care and healthy survival. One logical assumption is that people who experience little or no continuity with the future self may not aspire to control future health-related processes whereas people who experience much similarity or self-continuity with the future self are likely more motivated to work toward shaping a better survival. 

While previous research has demonstrated empirically the predictive value of the preceding sets of cognitive beliefs and perspectives in regard to well-being and physical and mental health-related processes and outcomes, findings have been drawn from the study of a wide range of ages. Thus, there is the question of whether the prognostic value of these belief systems continues into advanced old age. To date, the number of longitudinal studies of cognitive beliefs as predictors of longevity or as risk to mortality in old age is limited. To address this issue, longitudinal data obtained exclusively from samples of adults in advanced age are required. Accordingly, the purpose of our current research was to examine longitudinally the extent to which specific and select sets of cognitive beliefs are enabling, strengthening, or disabling with respect to long-term health, resilience, and longevity of older adults and have predictive value for all-cause mortality in advanced old age. The question is of increasing interest to health professionals and gerontologists for both practical and conceptual reasons.

In the section which follows, we review briefly the research literature that both explains and extends the assumptions underlying the theory and goals of concepts of BJW, FTP, and FSC and their potential for predicting longer survival/or reduced risk for mortality.

## 2. Conceptual and Theoretical Framework for the Research

The just world hypothesis and how it may relate to health and longevity is easily stated. Individuals have a strong need to believe that they live in a world where people generally get what they deserve. The belief that the world is just enables the individual to confront his/her physical and social environment as though they were stable and orderly. Without such a belief, it would be difficult for the individual to commit himself/herself to the pursuit of long-range goals or even to the socially regulated behavior of day-to-day life. Since the belief that the world is just serves such an important adaptive function for the individual, people are very reluctant to give up this belief, and they can be greatly troubled if they encounter evidence that suggests that the world is not really just or orderly after all [20]. According to Lerner and Miller's just-world theory, people who believe that the world treats them fairly may plan confidently for their future, expecting their lives to be orderly, meaningful, and controllable, foreseeing a positive future or viewing one's living situation as justly deserved and hence fair [21]. In turn, this expectation promotes mental health, meaning that the belief in a just world (BJW) can be seen as a “positive illusion” [22]. Indeed research links BJW to many indices of subjective well being including a greater purpose in life and commitment to planned healthy survival [23]. There is empirical evidence showing that individuals who strongly believe in a just world have been seen to experience less stress and more positive affect than individuals with a weaker BJW (e.g., [16, 24]).

The preceding conceptual underpinnings and the recent theory and research related to cognitive beliefs of a just world (BJW) lend weight to the proposed hypothesis of our present study that strong BJW beliefs about justice for the self portend positive social consequences and health-related benefits for the future. As such, individuals' strong cognitive beliefs about a just world (BJW) may be early predictors of their continued physical and mental well being at later stages of life and would serve to protect them against the stress associated with the challenges of later life. 

In the current study, we reason that the predictive power of BJW to enhance longevity derives uniquely from perceived justice and BJW beliefs. In light of the preceding discussion, our leading hypotheses for the current study were (1) that individuals' beliefs that the world is just to themselves (BJW-self) are particularly predictive of their longer term survival and longevity, and (2) that the associations between cognitive beliefs of a just world would be observed more powerfully among measures of *BJW for self only* (as distinguished from *BJW for others*). In essence, we reason that it is the perception of one's own, more so than other individuals', outcomes that would most powerfully predict longevity or possible risks of mortality; (3) that individuals' stronger levels of interpersonal trust and trust in the major communal institutions (as associated with their perceptions of justice in the BJW beliefs) are early predictors of their longevity, or conversely stronger levels of distrust in the major communal institutions would be associated with increased risk of mortality.

A related second goal of our current study was to examine the predictive value of other related cognitive perspectives such as future time perspectives not previously studied as predictors of mortality and longevity. On the basis of postulates derived from Carstensen's [11] theory on the influence of a sense of time on human development (also see [13, 25]) theory on individual differences in future self-continuity, we reason that while time eventually runs out for all individuals, individuals who hold more expansive and open-ended future time horizons or who foresee stronger self-continuity with the future self (compared to those who hold more limited future time horizons and less self-continuity with the future self) are less likely to discount the significance of future time and are more invested in self-preservation for the future. Accordingly, we hypothesize (4) that individuals' varying beliefs about future time left (FTP) and their beliefs about their ability to maintain self-continuity with the future (FSC) are critical markers or early predictors of longevity and of the risks of mortality.

While we acknowledge that the preceding associations between mortality and cognitive beliefs may not have been apparent in early and middle-age adulthood, our expectation is that the associations will be especially observable and relevant in late life functioning and will emerge as early markers or predictors of mortality or longevity.

## 3. Sampling Frame for the Study and Recruitment of Participants

Participants for the study were randomly recruited from the registry listings of four branch offices of community services and community organizations for seniors (Ministry of Health Services and Health Policy 1992), a governmental organization responsible at the time for social services and health policy in Southern Alberta. A sampling strategy with proportional stratification in function of geographical zone (metropolitan, urban, or rural) was used to ensure that the sample was representative of the general population of older adults living in three big cities and various rural areas in Southern Alberta. Participants came from three mid-sized cities (populations ranging from 170,000 to 300,000 individuals) and surrounding suburban and rural areas in Southern Alberta (Canada). It should be noted that various levels of community dwellings ranging from upper middle class private houses to low income apartment housing, and assisted living homes were included in the final recruitment.

Initially 760 brochures briefly describing the research were mailed on a staggered basis to seniors' households requesting individuals' participation with a one-time offer of a $30 gift certificate to compensate for their time. The purpose of the study was explained as an attempt to understand older individuals' beliefs, hopes, expectancies, and planned goals for the foreseeable future. By the end of eight weeks, responses were received from 132 individuals who volunteered their participation. The remaining 628 individuals were subsequently recontacted by mail, and of these 137 individuals who wanted additional information about the research were contacted by phone. Another 333 individuals agreed to participate in response to further advertising and a to a *second *and *third* “Call for Participants,” made 3 and 4 months later, bringing the total number of willing participants to 470. Eligibility criteria for the selection of participants included (1) being 65 years or older; (2) being able to understand, speak, and write English; (3) not having a diagnosis of cognitive dysfunction registered in the medical files; (4) being available for baseline assessments; (5) able to specify by name, address, phone number, and other relevant details, one or more family members or care-givers willing to serve as informants to the research team; (6) willing to sign a consent form.

### 3.1. Procedures

The initial interview with each of the 470 participants and their family members took approximately one-and-a-half hours, with interviews staggered over a period of 16 weeks. The majority of participants were interviewed in their homes in order to obtain baseline information about health status in relation to the chronic condition presented, to obtain participants' formal consent to participate and to arrange for their family member or primary care giver to contact us periodically concerning the participant's general progress. Typically, two family members for each participant contracted to be the informants. There were 62 husband-wife couple participants. We provided informants with postage-paid envelopes to contact us at regular intervals, as stated in the contact form, concerning the participant's health status (improving, stable, declining somewhat, seriously declining), and in the event of the participant's death, to provide the exact date of death. 

Within the first four months of the completion of baseline assessments for the study, a total of 30 participants withdrew their participation for a number of reasons, mostly because of the care-givers' reluctance to cooperate. Data are presented for the remaining 440 participants who, following baseline assessments, stayed the entire 6.5 years' course of the study. We wrote “thank you notes” and sent gift coupons to care givers at every wave of the study, as a token of appreciation of their continuing participation. There was no further attrition of participants during the course of the next 6.5 years. Ascertainment of mortality (date of death) was done solely on the basis of report of the informants. Family members preferred that we used this follow-up and contact procedure because it was more personal and private. However, as far as possible, we double checked dates of death against easily accessible local/provincial mortuary listings.

#### 3.1.1. Time Line for the Study

The study comprised 11 waves of data collection. Following a small pilot study, we conducted in 1995 on study procedures and assessment scales, baseline measures (wave 1) were obtained between September and December 2000 in a staggered way, followed by 10 subsequent waves of contacts with informants and/or participants to obtain “summary progress” data on participants. Contacts were made at approximately eight-month intervals (240 days apart) till early December 2007, approximately 6.5 years after baseline.

#### 3.1.2. Assessment of Cognitive Beliefs about a Just World, Interpersonal Trust and Control, and Perspectives on Future Time and Self-Continuity with the Future

Participants agreed to complete paper-and-pencil tests at their own pace, at home or in their place of study, and approximately 10 percent sought the help of research assistants to record their responses. 

At baseline, participants completed the following.

**Measure of Beliefs of a Just World (BJW)**

BJW were assessed with a scale originally developed by Lipkus and Siegler [9] but further improved by Bègue and Bastounis [23], in order to separate items of BJW pertaining to self from items of BJW pertaining to others. Participants rated on a scale of 1 (*strongly disagree*) to 6 (*strongly agree*) 8 items of BJW beliefs pertaining to the self. Sample items are “I feel that the world treats me fairly in life,” I feel that I get what I deserve,” “I feel that my efforts are noticed and rewarded,” I feel that people treat me with the respect I deserve,” “I feel that I earn the rewards and punishments I get.” Sample items from the BJW pertaining to others include “I feel that the world treats other people fairly,” “I feel that people get what they deserve,” “I feel that people get what they are entitled to get,” “I feel that when people meet with misfortune, they have brought it upon themselves.” Scores for BJW (self) and scores for the BJW (others) ranged from 8 to 48. We used the option of scoring the BJW-self and BWJ-others as continuous scales. Higher scores denote stronger BJW beliefs for self and others rated separately. Cronbach's alpha were .84 and .74 for the BJW-Self and BWJ-Others, respectively.

**Measure of Future Time Perspective (FTP)**

FTP was assessed with the future time perspective scale developed by Carstensen [26]. Participants rated on a scale from 1 (very untrue for me) to 7 (very true for me) the degree with which they agreed with each of 10 items. Sample items are “Many opportunities await me in the future,” “Most of my life still lies ahead of me,” “I expect that I will set many new goals in the future,” “My future seems infinite to me,” “There is plenty of time left in my life to make new plans,” “I have the sense that time is running out,” “As I get older, I begin to experience time as limited.” We used the option of scoring the FTP as a continuous scale in the first instance. Scores on this measure ranged from 10 to 70. The high scores represented a more expansive and open-ended future time perspective. Cronbach's alpha for the FTP scale was .82.

**Measure of Future Self-Continuity (FSC)**

FSC was assessed by means of an adapted psychometric measure of future self-continuity originally devised by Frederick [12] that used a single-item measure (i.e., how similar/connected are you to your past and future self for 5-, 10-, 15-, and 20-year intervals on a 1–100 scale?). To facilitate the comprehension of the concept of continuity, the individual's endorsement of similarity between present and future selves was presented pictorially by a range of five circles with* no overlap* to circles with *complete overlap*. The index of future self-continuity featured two questions on 7-point scale marked at each point by two circles that ranged from showing no overlap at one end of the scale to depicting almost complete overlap at the other end (see [27] for circles depicting no overlap to complete overlap), thus, making the measuring devise more concretely comprehensible to older adults. Participants first selected the pair of circles that best described how similar/connected they felt to the future self 15 years from now, and how much they cared about the future self. Subsequently, their responses were invited to two questions: “How connected do you feel to your future self 15 years from now?” (*not at all * = 0 to *very much* = 7). “How much do you care for your future self?” (*not at all * = 0 to *very much * = 7). We used the option of scoring the FSC scale (similarity/caring) as a continuous scale in the first instance. The index score of future self-continuity ranged from 7 to 35, and caring for the future self ranged from 7 to 35, assessed in terms of one continuous total index score ranging from 14 to 70. The high scores represented higher levels of future self-continuity/caring for future self. Cronbach's alpha for the FSC scale was .74.

**Interpersonal and Society Trust Measure**

Individuals' degree of interpersonal trust and trust in the surrounding public institutions that are perceived to represent justice and fairness were assessed by means of scale items adapted from the* The Rotter Trust Scale* [19]. As a first step in adapting the scale, a number of items were written using a 5-point Likert format, (1) strongly agree to (5) strongly disagree. An attempt was made to sample a wide variety of social objects so that a subject would be called upon to express his/her *trust *of outside agents, friends, and family members. Sample items include “In dealing with strangers one is better off to be cautious until they have provided evidence that they are trustworthy,” “Most elected public officials are not really sincere in their campaign promises”; “Most friends can be trusted to support you for life”; “I am able to share my innermost thoughts and feelings with family because of my trust in them”; “I do not like to reveal personal information to outside agents even when they claim to be helping me”; “I am wary of other people's motives” “I believe that most people are basically good and trustworthy”; “I am a private person and find it hard to trust people I do not know well.” In the final form of this scale, the 24 items selected were similarly balanced. Twelve items indicated *trust for agreeing,* and 12 items indicated *distrust for agreeing* (with the range of scores for both the trust items and distrust items being 12 to 60). A few filler items were included to disguise the true purpose of the scale. Cronbach's alpha for the trust scale and distrust scale were .77 and .72, respectively.

**Spheres of Control [18]**

The scale has little or no conceptual overlap with the BJW scale and, hence, was selected to provide an independent measure of control. The scale is comprised of 30 items with the three spheres of control (personal efficacy, interpersonal control, and sociopolitical control) each represented by 10 items each rated on a 5-point Likert scale ranging from “disagree” to “agree”. In the present research, we collapsed the scores across these three spheres of control because the results of our hypothesis tests were not affected by distinguishing between them. Specimen items for the personal efficacy scale, interpersonal scale, and sociopolitical scale, respectively, include “It's pointless to keep working on something that is too difficult for me”; “When I make plans I am almost certain to make them work”; “I have no trouble making and keeping friends”; “I find it easy to play an important part in most group or individual situations”. “In the long run, we as voters are responsible for bad government on a local or national level”; “It is difficult for people to have much control over things politicians do in offices.” The scoring of some items is reversed before summing the subset. A total index score for the 30 items was obtained with scores ranging from 30 to 150. Higher scores represent a more internal locus of control. Cronbach's alpha for the Control scale was .75.

**Measure of Self-Esteem (SEI: [28])**

This inventory was used to measure self-esteem as a global and stable disposition. The inventory has 10 items, 5 positively keyed and 5 negatively keyed. Each item is rated on a 4-point Likert type scale. Cronbach's alpha for the SEI scale was .88.

**Physical Function**

Physical Function was assessed by means of a single item taken from the physical function mobility index inquiring about one's ability (*yes/no*) to climb one flight of stairs without help. This one-item question was intended to seek information on physical fitness.

It should be noted that all measures administered to the participants were formatted in terms of language and structure appropriate for adults having a ninth-grade education. All paper-and-pencil tests and self-report measures used in the study were previously piloted on a volunteer group of 20 men and women aged 60 to 80 years. Subsequent modifications were made in the instructions and illustrations given for responding to the five-point ratings of test items. This procedure was undertaken to ensure that even those participants who were elderly and had relatively limited education could validly complete the measures.

#### 3.1.3. Assessment of Health Variables and Family Relationship Measures

Advanced old age is commonly associated with an increase in disabilities, higher rates of health care use, and higher need for social support. Thus, we felt the need to examine further fluctuations in risk of mortality after controlling for health-related covariates, which were assessed as follows:

**Survey of Number of Visits to Health Providers**

As a part of a separate survey, respondents listed the number of visits that they had paid in the previous year to health providers, including visits to family physicians, community health clinics, and emergency health units, including hospital visits.

**IADL Index of Disability**

We based this index on the self-reported ability for 12 daily living activities included in the IADL. Respondents were asked to indicate whether they had experienced limitations in 12 areas of functioning with responses coded (1) for *yes *and (0) for *no, *for example, able to use a telephone; boarding a bus without assistance; lifting or carrying groceries; personal grooming and personal hygiene care; doing light house work; taking medications; walking one block. All the items were summed to form one index of IADL, with scores ranging from 0 to 12. Cronbach's alpha for the IADL scale was .81.

**Measure of Satisfaction with Family and Social Support**

Social satisfaction was assessed with 10 items adapted from Zimet et al. [29]. Participants rated how satisfied they were with their social partners in general, and how satisfied they were with their family and relatives on a rating scale ranging from 1 (*very dissatisfied*) to 3 (*very satisfied*). Scores ranged from 10 to 30. Cronbach's alpha for the social support satisfaction scale was .89.

### 3.2. Data Analysis

We conducted all analyses using the *statistical package for social sciences *(*Version10*). We assessed the internal consistency of each trait scale with Cronbach's coefficient alpha and the association of the scales with each other and with other covariates by means of Pearson correlation coefficients. For all analyses, differences between survivors and decedents on the date of final censoring were assessed with *t*-tests and chi-square tests of association.

Cox proportional hazard models [30] were fitted to estimate the importance of each predictor of mortality. It is important to note that Cox regression, or the proportional hazards model, is a well-recognized statistical technique for survival analysis which is concerned with studying the time between entry to a study and a subsequent event such as death, as is the case in the present research. Cox regression has the advantage that it allows for the simultaneous exploration of the relationship between survival of persons and several explanatory variables (in our case variables such as cognitive beliefs, future time perspectives, control and trust, etc.). Of particular relevance and interest to our research is that the technique allows for age adjustment of the calculated hazard ratios across a wide range of ages, as was the case in our study. (See “What is a Cox model?,” by Stephen J. Walters, a Haywood Group plc publication, May 2003, accessible online at http://www.whatisseries.co.uk/whatis/pdfs/What_is_Cox_model.pdf); see also G.D. Garson, Statnotes, North Carolina State University, online at http://faculty.chass.ncsu.edu/garson/PA765/cox.htm) for details of statistical procedures for achieving adjustment for the effect of age as a covariate. The Cox regression model assumes that the death rate of the population depends on a continuous time variable, which in the present study was the interval between the Wave 1 assessment and date of death, calculated in terms of total number of days. 

In the present study, predictor variables included beliefs in a just society (BJW: self and others), future time perspective (FTP), future self-continuity (similarity/caring) perspective (FSC), demographic variables, and health and physical functioning variables. The initial risk ratios (IRRs) (frequently referred to as hazard ratios) are calculated by exponentiating the beta weight for the predictor [31], and regressions are expressed in risk ratios and 95 percent confidence intervals. All Cox regression hazard ratios (IRRs) presented in the present analyses were first adjusted for age. In subsequent analyses, we controlled for three health-related covariates (see [3, 5, 32, 33] for significance of studying health variables).

## 4. Results

Results are reported in two sections corresponding to our two main research questions. In the first section, we examine key differences between survivors and decedents in baseline characteristics of participants. In specific, we conducted Cox regression analysis of risk rations of mortality to estimate the relative risk of death and the importance of each predictor of mortality for the whole sample. All variables entered in the Cox regression analysis were entered as continuous variables for these analyses. In the second section, we present additional analysis of data where we were unable to derive easily interpretable results from using Cox regressions of continuous variable data. Subsequently, we analyzed the data using categorical variables that we considered were appropriate for the otherwise continuous variables. 

### 4.1. Key Differences and Associations between Survivors and Nonsurvivors (Decedents)

Four hundred and forty participants were followed starting in December 2000 when baseline assessments were completed. As of December 2007, after an average of 6.5 years of observation one hundred and forty (32%) deaths had occurred, and 300 (68%) survived.  Table 1 provides crude data at baseline on the two subgroups of decedents and survivors. Those who died during the study period were somewhat older. Among health-related variables, the respondents in the two groups were quite different with respect to physical function scores, but not with respect to number of medical visits reported at time of baseline assessment, or in respect of number of disabilities reported on the IADL index (see Table 1). Survivors compared to decedents had significantly higher scores on measures of beliefs in just world (BJW-Self), on the future time perspective (FTP) and future self-continuity/caring for the future self (FSC) perspective scale. By contrast, decedents compared to survivors had significantly higher scores on the measure of distrust on the interpersonal and society scale and on the control scale.

First, we examined the intercorrelations of measures of beliefs in a just world (BJW; self and others), future time perspective (FTP), future self-continuity perspective (continuity/caring for future self), trust (agreement with trust items and agreement with distrust items) and control with one another, and with demographic variables (age and education), and baseline indicators of number of medical visits, IADL disability, and satisfaction with social support. As expected, the two scales of BJW were positively related to each other, with correlations ranging from .41 to .64; *P* < .001. Overall, the BJW-self scale, the FTP scale, and FSC scale were positively correlated with one another, with correlations ranging from .34 to .68; *P* < .001. In general, the BJW, FTP, FSC, and trust measures were not correlated significantly with demographic variables of age and education. The one notable exception, however, was that the distrust measure was significantly correlated with age and education, with correlations ranging from .34 to .39; *P* < .001 (Note: the detailed table of correlations is available from the authors on request).

#### 4.1.1. Psychometric Information on Two Dimensions of BJW (Self/Others), Two Dimensions of Future Time Perspectives (Time Left/Self-Connected with Future), Trust/Distrust and Measures of Self-Esteem and Control

Table 2 provides psychometric information on the predictor variables for mortality risks selected for the research.

Examination of the mean scores for the various scales for which baseline data were obtained shows that the mean score of the control scale (80.1) was quite elevated when considered within the context of validity norms outlined in Paulhus [18]. However, the mean scores for the BJW, FTP, FSC. Trust and Distrust scales were consistent with means reported for these scales elsewhere. The internal consistencies of the various trait measures as reported in Table 2 are consistent with and converge appropriately with internal consistencies reported for the various scales elsewhere (see [10, 12, 19, 25]).

When Cox proportional hazard models *adjusted for age* [30] were fitted to estimate the relative risk of death and the importance of each predictor of mortality for the whole sample of 440 participants, the findings showed that BJW (self), FTP, and FSC were positively and significantly related to* reduced risk of mortality*. In other words, scores on these measures were *inversely *related to risk of mortality, and associated with a significantly *reduced* risk of mortality. As seen in Table 3, BJW (others), trust, self-esteem, and social support satisfaction were associated with a rather weak or marginal reduction in risk of mortality. By contrast, distrust was related to a significantly* increased risk of mortality.*

### 4.2. Results of Additional Analyses

We conducted a hierarchical regression analysis (*N* = 440) in order to examine further the relative contribution of the variables to risk of mortality (Table 4).

This analysis revealed the ability of BJW-self to predict risks of mortality over and above other predictor variables. As seen in Table 4 (Model 1 Model 2), results clearly show that adding the cognitive beliefs of BJW (self) variable separately contributed to the explanation of variance in mortality risks significantly and uniquely, over and above that offered by the three variables of FTP, FSC, and distrust. In other words, the results in Table 4 confirm that BJW (self) predicts an association with significant reduction in mortality risks independent of the other predictor variables. This rules out the sceptical view that the ability of BJW-Self to predict outcomes is an artifact of correlations with other factors previously associated with outcomes of BJW, for example, control or trust.

### 4.3. Comparisons of Extreme High Scorers and Extreme Low Scorers Using Trichotomization

Additionally, we conducted a preliminary examination of how deaths were distributed across low, average, and high trait standard scores. Our inquiry suggested possible violations of the Cox proportional hazards assumption. The proportionality assumption is violated when the relative risk of the outcome does not change in the same manner for equivalent changes in the levels of a risk factor or covariate [34]. Categorical variables may then be appropriate for an otherwise continuous variable [34] and predictors may be trichotomized based on broad cut-off points [35]. Although trichotomization may reduce statistical power, it has the possible advantage that trichotomized domain scores will be more readily interpretable [35]. Accordingly, following scores on each of the predictor variables (converted to standard scores), tertiles were computed for each of the predictor variable subscale measures. We plotted survival curves showing specifically and with greater focus on the relationship of predictor variables to mortality, with survival rates plotted for *extreme high scorers* (*at 67th percentile and above*) and *extreme low scorers *(*at 33rd percentile and below*).

**Figure 1:**
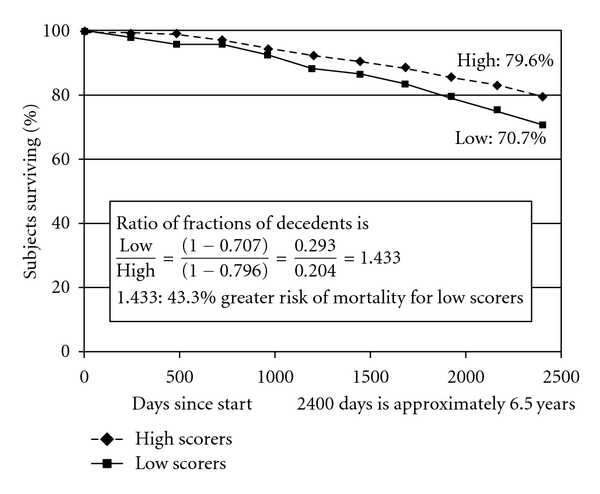
Survival in the groups with high and low scores on *BJW *(*self*) over approximately 6.5 years.

**Figure 2:**
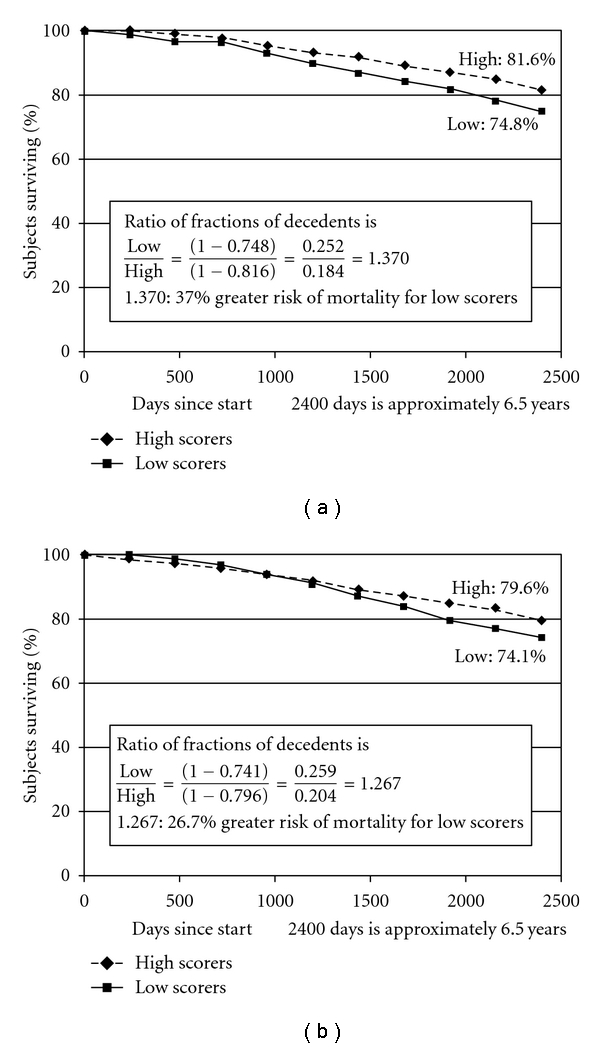
(a) Survival in the groups with high and low scores on *future time perspective* over approximately 6.5 years. (b) Survival in the groups with high and low scores on *future self-continuity* over approximately 6.5 years.

**Figure 3:**
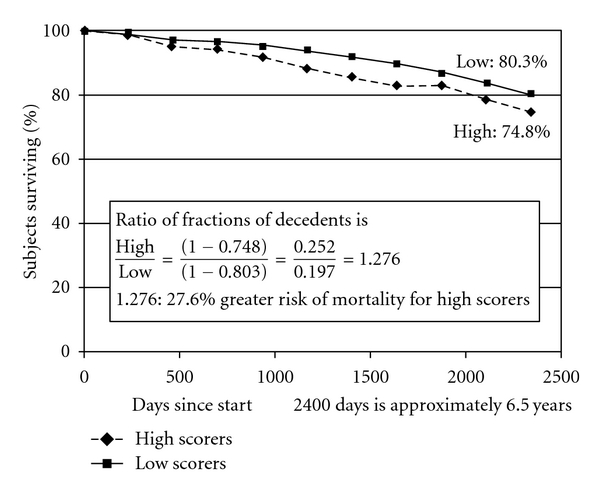
Survival in the groups with high and low scores on *Distrust* over approximately 6.5 years.

Four survival curves of particular interest and relevance to our research hypotheses are portrayed in Figures 1 to 3.

Figure 1 showing the survival curve for beliefs in a just world (BJW-self) suggests that individuals with *extreme high scores* (at 67th percentile and above) on this dimension are at significantly *reduced* risk for mortality compared to individuals with *extreme low scores* (at 33rd percentile and below). The percentages for those who survived in the two groups are 79.6% and 70.7% percent, respectively, indicating a 9% difference in mortality. The ratio of fractions of decedents to survivors (1 − 0.707)/(1 − 0.796) = 1.433 (see Figure 1) suggests a 43.3% *lower* risk of death for *extreme* high scorers compared to *extreme* low scorers. The findings from the survival curve presented in Figure 1 further confirm the predictive ability of this measure seen earlier in Table 3 where BJW-self was used as continuous variable in the Cox regression analyses. 

Figure 2(a) showing the survival curve for future time perspectives (FTPs) suggests that individuals with *extreme high scores* on this dimension (at 67th percentile and above) are at a significantly *reduced* risk* for mortality* compared to individuals with *extreme low scores* (at 33rd percentile and below). The percentages for those who survived in the two groups are 81.6% and 74.8% percent, respectively, indicating a 7% difference in mortality. The ratio of fractions of decedents to survivors (1 − 0.748)/(1 − 0.816) = 1.370; (see Figure 2(a)) suggests a 37.0% *reduced risk of death* for *extreme high scorers* compared to *extreme low scorers*. The findings from the survival curve presented in Figure 2(a) further confirm the predictive ability of this measure seen earlier in Table 3 where future time perspective was used as continuous variable in the Cox regression analyses. 

Figure 2(b) showing the survival curve for future self-continuity perspectives (FSCs) suggests that individuals with *extreme high scores* (at 67th percentile and above) on this dimension are at a significantly *reduced* risk for mortality compared to individuals with *extreme low scores *(at 33rd percentile and below). The percentages for those who survived in the two groups are 79.6 and 74.1 percent, respectively, indicating a 5.5% difference in mortality. The ratio of fractions of decedents to survivors (1 − 0.741)/(1-0.796) = 1.267 (see Figure 2(b)) suggests a 26.7% *reduced risk of death* for *extreme high scorers* compared to *extreme low scorers.* The findings from the survival curve presented in Figure 2(b) further confirm the predictive ability of this measure seen earlier in Table 3 where the future self-continuity perspective measure was used as continuous variable in the Cox regression analyses. 

Conversely, the survival curve for distrust (Figure 3) suggests that individuals with *extreme high scores* (at 67th percentile and above) on this dimension, compared with those with *extreme low scores* (at 33rd percentile and below), are at a significantly *increased risk for mortality.* The percentages for those who survived in the two groups are 80.3% and 74.8%, respectively, indicating a 5.5% difference in mortality. The ratio of fractions of decedents to survivors (see Figure 3) (1 − 0.748)/(1 − 0.803) = 1.276 suggests a 27.6% *higher risk of death* for *extreme high scorers* on distrust, compared to *extrem*e low scorers. The findings from the survival curve presented in Figure 3 further confirm the predictive ability of this measure seen earlier in Table 3 where the distrust measure was used as continuous variable in the Cox regression analyses. 

To sum up the results, when the tertile split analysis was done, BJW (self), future time perspectives (FTPs) and future self-continuity perspectives (FSCs) were found to be differentially associated with mortality risks. When BJW (self) scores, future time perspective scores, and self-continuity or similarity with the future self scores were *extremely low *(33rd percentile and below), *mortality risk was significantly greater*, compared to *extremely high *scores on these measures (67th percentile and above). In other words, high scores on these measures represented reduced risk to mortality. However, the variable of distrust revealed a somewhat contrasting pattern in the tertile analysis. When distrust scores were *extremely low* (33rd percentile and below), *mortality risk was significantly lower*, compared to *extremely high distrust scores* (67th percentile and above) which were associated with *an increase in mortality risk*.

### 4.4. Controlling for Health-Related Variables

In a separate set of regression analyses, we explored the influence of three health-related covariates (Table 5). These analyses were intended mainly to see whether differences in cognitive predictors of mortality would be maintained after controlling for three health-related covariates assessed at baseline: (1) number of visits in the previous year to medical practitioners and health providers, (2) total index score for the measure of disability (IADL) in daily life activities, and (3) total score for self-rated satisfaction with family and social support. Initially, these health-related covariates were controlled for one at a time, using a Cox regression analysis procedure. However, the results of the Cox regression analyses were ambiguous. We then re-ran the analysis using multiple regression, adjusting for the covariates (Table 5).

As seen in Table 5, the negative beta values for beliefs in a just world, future time perspective, future self-continuity, and social support satisfaction, and the significant *F *values observed in the multiple regression analysis are consistent with the *direction of reduced risk ratios* shown in the relative risk (RR) and 95% confidence interval (CI) model (Table 3). Similarly, after adjusting for the covariates, the positive direction of the beta values and the significant *F *values for distrust in the multiple regression analyses is consistent with the *direction of increased risk ratios* for this variable in the relative risk (RR) and 95% CI model. Thus, overall, the relationship between cognitive beliefs about a just world, future time perspectives, and risks for mortality were maintained after adjusting for the health-related covariates. In other words, the direction of the association we saw in the earlier Cox regression analyses (Table 3) between scores on cognitive beliefs, future perspectives, distrust, and risks for mortality remained unchanged after adjusting for the health-related covariates. 

In further analyses, we found no statistically significant interaction effects among the sociodemographic variables and health variables relating to the prediction of mortality.

## 5. Discussion and Interpretation of Results

### 5.1. Beliefs in Just World (BJW-Self)

Consistent with our hypothesis, our findings from the Cox regression analysis of the predictor variable of BJW revealed that BJW is positively related to survival. Additionally, our findings based on a hierarchical regression analysis of a two-model design show that the variable of BJW (self) had stronger ability than all other cognitive variables explored in this research to predict mortality risk. These findings lend weight to recent theory and research suggesting that the predictive power of BJW-self seems to derive uniquely from perceived justice and that most persons have a strong need to perceive the world to be just, a belief system that conceivably serves to protect their health and survival [10, 14, 23]. Our findings that high levels of beliefs about justice and fairness are early predictors of longer survival are consistent with our hypothesis. These findings advance and extend the assumptions of earlier researchers' work on perceived justice and fairness [9, 10] as linked to better health and well being. Our findings show that individuals' concern that the world is just to themselves, as distinguished from others, is particularly predictive of their healthy survival. Along with earlier researchers, we speculate that BJW for self contributes to a larger extent to individuals' psychosocial adjustment, providing them with useful resources in times of stress, and correspondingly becomes linked with reduced risks of mortality. In our study, BJW (others) was not differentially linked with mortality risks. However, we cannot conclude that BJW-Self alone generates the link with reduced risk of mortality. Consequently, the mortality correlates with BJW in the spheres of self and others remain to be determined more fully.

### 5.2. Future Time Perspective

Overall we predicted that while time is finite and limited for all, an increased or more expansive or open-ended future time perspective would reduce the risk of mortality, based on a logical assumption that a more expansive view on time left may promote valuation of future plans to safeguard the future self and protect it against risks of mortality. Consistent with our hypothesis, our findings from the Cox regression analysis (adjusted for age) show that an expansive future time perspective is associated with a reduced risk of mortality for our older sample. Our findings lend support to earlier postulates that the subjective sense of time plays an essential role in human motivation, and gradually time left becomes a better predictor than a range of other cognitive variables that contribute to behavioral and psychological processes at work in old age [11]. The present direction of findings advances and extends assumptions of future time perspectives proposed by previous researchers (e.g., [12]) according to whom individuals' perceptions of remaining time to live determines their potential for evaluating and shaping future time and future prospects concerning identity and health. Extrapolating from our findings, we speculate that incorporating an expansive time horizon influences individuals to pay relatively greater attention in the time left to make pragmatic and more practical decisions concerning a healthier future life. Additionally, we speculate that individuals in our sample who showed more expansive or open-ended time horizons at the time of baseline assessment would have already maximized attempts to preserve and protect future personal health and survival. However, our findings suggest that older adults' perception of time left is linked rather nonspecifically with risks of mortality. It remains for future researchers to disentangle the specific psychological processes that link limited horizons of time left with increased risk of mortality.

### 5.3. Future Self-Continuity

Overall, we predicted that increased future self-continuity and caring for future self would reduce the risk of mortality, based on a logical assumption that increased future self-continuity perceptions may promote the valuation of future plans to safeguard the future self and to protect it against risks of mortality. Consistent with our hypothesis, our findings from the Cox regression analysis (adjusted for age) show that future self-continuity as a variable is associated with reduced risk of mortality. *Extreme high scorers* on future self-continuity and caring for the future (i.e., individuals who perceived greater similarity between their present and future self), compared with *extreme low scorers* (i.e., individuals who perceived minimum similarity between their present and future self) had a significantly reduced risk of mortality (37%). Although the present results cannot specify the causal direction of the association between future self-continuity and reduced risk of mortality nor specify the health-related processes at work to explain this association, the research findings provide initial evidence that high similarity and high self-continuity with future self is a significant predictor of longevity. Overall, our findings fit well with the philosophical analysis by Parfit [36] that individuals who feel similar to their future selves are more likely to make more prudent decisions for the future, including health-related decisions, because of their perceived close connectedness to the future self. Alternatively, we speculate that individuals who perceived their current physical and mental health to be stable at the time of baseline assessments were also able to identify better with their future self as being one of healthy survival and, therefore, worth protecting. Many questions remain. Of particular interest, in the context of a longitudinal study, is the initial evidence that behavioral differences in longevity are driven differentially by perceived future self-continuity.

### 5.4. Trust/Distrust

Our findings from the Cox regression analysis, showing that high distrust predicted a significantly increased risk of mortality, are consistent with our hypothesis. These findings are not at variance with earlier theoretical proposals suggesting that historically older adults have had difficulty in accepting the status quo as defined by younger authorities in the social system and may tend to be more distrusting of those authorities. Our findings suggest that extreme high levels of distrust which typified some individuals in our sample, compared with extreme low levels of trust, increased the risk of mortality by 27.6 percent. Overall, the Cox regression analysis revealed that distrust is empirically predictive of an increased risk of mortality. We speculate that high levels of distrust may be analogous to a form of pathological social isolation or breakdown in communications that leads to increased risk for physical and psychological disease [37, 38]. Conceivably distrust becomes linked with a shorter life span.

### 5.5. Study Limitations

Despite the novelty of our data, and thus the importance of their contribution to cognitive etiology of health-related processes such as survival, mortality, or longevity, our study was subject to the limitation that the sample was a volunteer one. Our cognitive scale measures of beliefs in a just world, and other measures exploring individuals' perspectives on future self-continuity and future time, trust and distrust were self-report measures and subject to the biases associated with self-report measures. The preceding sources of unreliability of measurement could potentially both reduce or increase the proportion of variance in the outcome variables for which our models were able to account. However, we are confident that we had a sufficiently large and representative sample of urban and rural older adults, and the measures we used showed high internal consistency.

### 5.6. Conclusions

Our study provided novel indications that cognitive belief systems and future time perspectives of older adults may have much to contribute to increased or reduced mortality risks in late life. Thus, we suggest that basic cognitive beliefs and future time perspectives that were examined in the present research for their association with increased or reduced risk for mortality are an important direction for future research. It remains for future researchers to try to integrate these various cognitive factors into some kind of theoretical frameworks to gain a precise appreciation of how these factors may be causally linked with longevity.

### 5.7. Practical Implications for Clinical Practice

Extrapolating from the findings of the study concerning the association between beliefs in a just world and the potentials for a longer survival, it may be reasonable to postulate that the findings have possible implications for clinical practice with older adults who may be dealing with traumatic life events such as life threatening illness, loss of loved ones, and other natural disasters. It is conceivable that these individuals may, as a result of adverse and stressful experiences, feel an abrupt decline in their beliefs in a just world or a decline in their faith or trust in interpersonal connectedness. It is suggested that clinicians be particularly mindful of clients who score extremely low on measures of BJW and future time perspectives. Conceivably, they are at greater risk of mortality. Similarly, those individuals scoring extremely high on measures of distrust and control may be at greater risk of mortality. By exploring with older clients their beliefs about a just world and their trust or faith in interpersonal connectedness, geriatric service providers and psychologists may be able to assist clients to develop a realistic self-profile of their sources of life strengths in relation to self-growth, resilience, and control over every day life situations. Thus, clinicians may be able to assist elderly clients to draw on internal sources of strengths, such as reflecting on their beliefs about a just world and their beliefs about trusting others, as a means to strengthening and enhancing future self-continuity. Based on our findings suggesting that individuals' strong beliefs in a just world and positive perspectives regarding future time may possibly protect them against risk to mortality, geriatric service providers are encouraged to engage in a dialogue with their clients on these themes. Such dialogues may not only serve to act as buffers for clients against stress and anxiety about the future, but may also assist clients in building up greater motivation for living.

From a pragmatic standpoint, we postulate that interventions aimed at strengthening individuals' beliefs in a just world at an earlier age, and encouraging future-self continuity in the earlier adulthood years, might motivate people to care more for their potential person in the later years, and conceivably keep people healthier longer and thereby reduce risk of mortality. Similarly, communication programs that foster interpersonal and institutional trust or help older adults combat high levels of distrust (which in our research were associated with significantly increased risk of mortality) may be of value to a longer survival.

## Figures and Tables

**Table 1:** Baseline characteristics of participants who survived (survivors) or died (decedents) Total *N* = 440.

Characteristics (range of scores)	Survivors *N* = 300 (68%)	Decedents = *N* = 140 (32%)	*P* Value
Age, years	74.4 (6.3)	78.6 (6.5)	<.001
Education, years	14.1 (3.7)	13.9 (3.0)	.339
Women 66.0% (291) Men 34.0% (149)	195; 67% 102; 69%	96; 33% 47; 31%	<.001*
Beliefs in just world (BJW: self) (8–48)	32.8 (4.1)	20.4 (4.7)	<.001
Beliefs in just world (BJW: others) (8–48)	22.7 (2.1)	21.8 (3.0)	.461
Future time perspective (FTP) (10–70)	36.8 (6.1)	29.9 (7.6)	<.001
Future self-continuity (FSC)—15 yrs (14–70)	32.0 (3.5)	24.0 (2.1)	<.001
Trust: interpersonal and society (12–60)	33.1 (5.1)	29.5 (6.1)	<.05
Distrust: interpersonal and society (12–60)	27.5 (5.1)	39.9 (6.2)	<.001
Control (30–150)	69.6 (4.8)	67.0 (5.9)	.239
High satisfaction with social support (percentage)	61%	48%	<.001
Number of medical visits in preceding year, at baseline:	5.9 (2.1)	6.1 (2.8)	.139
Self-esteem (10–30)	16.8 (5.2)	15.9 (6.9)	.239
High physical functions score (percentage)	31%	22%	<.001
IADL disabilities (12 items)	5.00 (2.0)	6.00 (2.7)	.330

Note: All data are presented as mean ratings and (standard deviations) unless otherwise indicated. *P* values are based on *t*-tests; *denotes variables where *P* values are based on *χ* ^2^ tests of association.

**Table 2:** Psychometric information on beliefs in just world (self and others) and other related future time perspective measures and trust measures.

Measures	Mean	(SD)	*α*
Beliefs in just world (BJW: self) (8–48)	36.6	(6.1)	.84
Beliefs in just world (BJW: others) (8–48)	25.7	(5.8)	.74
Future time perspective (FTP) (10–70)	39.9	(5.2)	.82
Future self-continuity (FSC)—15 yrs (14–70)	34.8	(6.1)	.74
Agreement with trust items: interpersonal and society (12–60)	37.6	(5.3)	.77
Agreement with distrust items: interpersonal and society (12–60)	50.2	(6.9)	.72
Control (30–150)	80.1	(10.6)	.75
Social support satisfaction (10–30)	22.4	(3.82)	.89
Self-esteem (10–40)	23.9	(3.1)	.88

Note: The alpha denotes the coefficient alpha, a measure of internal consistency.

**Table 3:** Relative risk of death associated with beliefs in just world and other related future time perspective measures and trust measures (*N* = 440) (Range of scores).

Measures	Relative risk	95% Confidence intervals
Beliefs in just world (BJW: self) (8–48)	.798	.706, .892
Beliefs in just world (BJW: others) (8–48)	1.003	.996, 1.014
Future time perspective (FTP) (10–70)	.842	.817, .867
Future self-continuity (FSC)—15 yrs (14–70)	.865	.819, .913
Agreement with trust items: interpersonal and society (12–60)	.970	.940, 1.011
Agreement with distrust items: interpersonal and society (12–60)	1.049	.940, 1.158
Control (30–150)	1.005	.996, 1.034
Social support satisfaction (10–30)	.945	.929, .969
Self-esteem (10–40)	.970	.940, 1.011

**Table 4:** Hierarchical regression analysis (*N* = 440) showing ability of BJW-self to predict mortality over and above control, interpersonal and society distrust, future time perspective, and future self-continuity perspective.

Variable	Model 1		Model 2	
	*β*	*t*	*β*	*t*
Control	.061	.75	.065	1.05
Overall distrust	.459	6.18**	.399	4.44*
Future time perspective	.559	6.32**	.456	5.88**
Future self-continuity	.479	4.99*	.407	4.69*
BJW-self			.579	6.92**
Adjusted *R* ^2^	.475		.523	
*F* change	41.86**		18.14**	

*P <.05.

**P <.001.

**Table 5:** Relative risk of death associated with cognitive beliefs, future perspectives, and related traits after adjusting for three covariates entered in the multiple regression analysis.

Trait	*β*	*F*	Multiple *R*	*R* ^2^	Risk ratio model	
					RR	95% CI
Covariates combined	.54	6.55**				
BJW (Self)	−.48	5.91**	.51	.26	.832	.809, .855
BJW (Others)	.15	<1.00	.24	.06	1.004	.990, 1.018
FTP (Future time)	−.41	5.21**	.34	.12	.852	.809, .895
FSC (Future similarity)	−.31	4.27**	.28	.08	.842	.816, .868
Trust: agreement	−.18	2.02*	.20	.04	.976	.962, .990
Distrust: agreement	.37	4.57**	.26	.07	1.048	1.024, 1.072
Self-esteem	−.16	<1.00	.20	.04	.998	.964, 1.032
Social support satisfaction	−.29	2.01*	.17	.03	.935	.907, .963
Control	.21	2.11*	.14	.02	1.005	.996, 1.034

Total				.72		

*P < 05; **P < .001; Degrees of freedom for *F* = 12,424.

Covariates include: participants' (1) number of visits to health providers at baseline, (2) IADL index score for disability at baseline, and (3) satisfaction with family and friends support. Note: covariates were entered simultaneously. Relative risk ratio (RR) and confidence intervals (CIs) were determined after means were adjusted for covariates.
